# Neutrophil gelatinase-associated lipocalin (NGAL) predicts the occurrence of malaria-induced acute kidney injury

**DOI:** 10.1186/s12936-016-1516-y

**Published:** 2016-09-09

**Authors:** Marlies E. van Wolfswinkel, Liese C. Koopmans, Dennis A. Hesselink, Ewout J. Hoorn, Rob Koelewijn, Jaap J. van Hellemond, Perry J. J. van Genderen

**Affiliations:** 1 Harbour Hospital and Institute for Tropical Diseases, Rotterdam, The Netherlands; 2 Department of Medical Microbiology and Infectious Diseases, Erasmus MC and Harbour Hospital and Institute for Tropical Diseases, Rotterdam, The Netherlands; 3 Department of Internal Medicine, Erasmus MC, Rotterdam, The Netherlands

**Keywords:** Malaria, *Plasmodium falciparum*, Acute kidney injury, NGAL, KIM-1

## Abstract

**Background:**

Acute kidney injury (AKI) is a frequently encountered complication of imported *Plasmodium falciparum* infection. Markers of structural kidney damage have been found to detect AKI earlier than serum creatinine-based prediction models but have not yet been evaluated in imported malaria. This pilot study aims to explore the predictive performance of neutrophil gelatinase-associated lipocalin (NGAL) and kidney injury molecule-1 (KIM-1) for AKI in travellers with imported *P. falciparum* infection.

**Methods:**

Thirty-nine patients with imported falciparum malaria from the Rotterdam Malaria Cohort with available serum and urine samples at presentation were included. Ten of these patients met the criteria for severe malaria. The predictive performance of NGAL and KIM-1 as markers for AKI was compared with that of serum creatinine.

**Results:**

Six of the 39 patients (15 %) developed AKI. Serum and urine NGAL and urine KIM-1 were all found to have large areas under the receiver operating characteristics curves (AUROC) for predicting AKI. Urine NGAL was found to have an excellent performance with positive predictive value (PPV) of 1.00 (95 % CI 0.54–1.00), a negative predictive value (NPV) of 1.00 (95 % CI 0.89–1.00) and an AUROC of 1.00 (95 % CI 1.00–1.00).

**Conclusion:**

A good diagnostic performance of NGAL and KIM-1 for AKI was found. Particularly, urine NGAL was found to have an excellent predictive performance. Larger studies are needed to demonstrate whether these biomarkers are superior to serum creatinine as predictors for AKI in *P. falciparum* malaria.

**Electronic supplementary material:**

The online version of this article (doi:10.1186/s12936-016-1516-y) contains supplementary material, which is available to authorized users.

## Background

Malaria-induced acute kidney injury (AKI) is an important contributing factor to the high mortality of severe malaria [1]. In endemic regions, AKI occurs in up to 40 % of adult patients with severe *Plasmodium falciparum* infection and it is associated with high mortality [2–5]. Outside endemic countries, AKI is also a frequently encountered complication of imported *P. falciparum* infection. In a recent study in travellers with imported *P. falciparum* infection, AKI, defined according to the criteria set by the Kidney Disease: Improving Global Outcomes (KDIGO) AKI Work Group [6], was seen in 39 (8 %) of all 485 patients and in 23 (38 %) of the 61 patients with severe malaria [7].

Although the KDIGO classification allows for an early detection of changes in glomerular filtration rate (GFR), the use of serum creatinine-based prediction models for AKI has limitations. Substantial rises in serum creatinine are only observed 48 to 72 h after the initial injury to the kidney, and, because of factors such as enhanced tubular creatinine secretion, significant renal injury can occur without an important rise of serum creatinine [8]. This is illustrated by a recent study in which 12 (31 %) of 39 patients with malaria-induced AKI had a normal serum creatinine at presentation [7]. Because structural kidney injury precedes functional injury, a marker of structural kidney damage could theoretically detect AKI much earlier, in a way that has been compared to the role of troponin in the detection of myocardial injury [9].

A promising marker of structural kidney damage in AKI is neutrophil gelatinase-associated lipocalin (NGAL), also known as lipocalin 2. This protein was initially discovered in activated neutrophils and was later found to be produced by many tissues, including renal tubular epithelial cells. Under physiologic conditions, NGAL is present at very low concentrations in the urine and plasma [10–12]. Studies in animals showed NGAL to be one of the most upregulated genes in the kidney very early after AKI. As a consequence of the induction of NGAL-expression in the distal nephron and reduced proximal tubular re-uptake due to tubular damage, both urine and plasma NGAL concentrations rise quickly in the event of AKI [10, 11]. The value of NGAL as a predictor of AKI has been established in a large number of studies, predominantly performed in critically ill patients, after cardiac surgery and after kidney transplantation [11, 13, 14]. In studies in endemic regions, urine NGAL has been shown to rise with increasing severity of AKI in severe malaria [2] but was not superior to creatinine in predicting in hospital requirement for renal replacement therapy (RRT) [15]. However, NGAL has not yet been evaluated as a marker for AKI in patients with imported malaria.

Another frequently used biomarker for kidney injury is kidney injury molecule-1 (KIM-1). KIM-1 is a transmembrane protein in tubular kidney cells that is undetectable in plasma of patients with undamaged kidneys. In damaged tubular epithelial cells undergoing dedifferentiation and proliferation, KIM-1 is induced and can become detectable in plasma and urine [16]. The exact function of KIM-1 is unknown and reports have suggested both a protective and a damaging function of the molecule [16]. Urinary KIM-1 concentrations were found to increase much earlier than blood urea nitrogen and plasma creatinine in studies in which proximal tubule injury was induced by cadmium or ischemia [8, 17, 18]. Its performance as an early marker for AKI has been evaluated in several clinical settings, including contrast-induced nephropathy [19–21] and sepsis [20], but not during malaria.

The aim of this pilot study was to explore the predictive performance of NGAL and KIM-1 in AKI in travellers with imported *P. falciparum* infection.

## Methods

### Patients

The Harbour Hospital is a 161-bed general hospital, located in Rotterdam, The Netherlands. It also harbours the Institute for Tropical Diseases, which serves as a national referral centre. The Rotterdam Malaria Cohort consists of all patients diagnosed with malaria at the Institute for Tropical Diseases since 1998. Of all these patients, anonymized demographic, clinical and laboratory data are routinely collected and stored in an electronic database. On 1 January, 2015, the Rotterdam Malaria Cohort comprised 690 cases of imported malaria, of which 491 were caused by *P. falciparum.*

The present pilot study focussed on the diagnostic accuracy of serum NGAL (sNGAL), urine NGAL (uNGAL) and urine KIM-1 (uKIM-1) concentrations as predictive biomarkers for AKI. Thirty-nine patients with imported falciparum malaria from the Rotterdam Malaria Cohort with available serum and urine samples at their initial presentation were included. Ten patients with imported *P. falciparum* met the criteria for severe malaria. The findings were compared with the performance of serum creatinine as a marker for AKI in falciparum malaria.

### Laboratory investigations

All laboratory parameters were measured on admission with routine procedures, as described before [22]. The standard procedure to diagnose malaria comprised a quantitative buffy coat (QBC) analysis, a rapid diagnostic test (RDT) for malaria antigens (Binax NOW^®^ Malaria Test Binax, Inc, Maine, USA), and thick and thin blood smears using freshly collected blood specimens from finger pricks. The RDT and the QBC analysis were performed according to the manufacturer’s instructions [23, 24]. QBC capillaries were examined independently by two technicians by microscopic analysis of two complete rows of the region between the bottom of the capillary and the polynuclear leukocyte layer using an Olympus BX-60 fluorescence microscope equipped with UV-filter, 50× objective and 12.5× oculars (total magnification 625×).

Measurements of sNGAL, uNGAL and uKIM-1 were done using residual patient material. Samples of serum and urine had been collected at initial presentation and stored at −80 °C until analysis. sNGAL with a maximum storage time of 2 years and 9 months, during which stability of NGAL and KIM-1 levels can be assumed [25]. uNGAL and uKIM-1 were quantitatively determined with commercially available sandwich ELISA kits (NGAL Quantikine and KIM-1 Quantikine kits from R&D Systems, Inc, Minneapolis, USA).

### Definitions

#### Acute kidney injury

AKI was defined using the KDIGO criteria, which include three stages of progressive renal dysfunction (see Additional file 1) [6]. Since patients may present with renal dysfunction without a known baseline serum creatinine, a classification based on a change of creatinine or estimated GFR (eGFR) from baseline poses a significant limitation. To circumvent this problem, the following method, which was previously described as a suitable method by the ADQI Group [26], was used: in patients without previously measured creatinine concentrations, a normal premorbid renal function was assumed. A baseline, creatinine concentration was then calculated, using the four-variable modification of diet in renal disease (MDRD) formula with an assumed normal eGFR. These calculated baseline values were used to estimate the change of renal function during infection (see Additional file 2).

#### Severe malaria

Patients were considered as having severe falciparum malaria if they met the recently updated World Health Organization (WHO) criteria for severe malaria on admission or during hospitalization (see Additional file 3) [27].

### Statistical analysis

All data were extracted from the original secured database and reviewed for inconsistencies and missing values. For statistical analysis, IBM Statistical Package for the Social Sciences (SPSS) version 22 (IBM Inc, Chicago, IL, USA) was used. Differences between the groups having AKI or no AKI were analysed using the Fisher’s exact test or Chi square test for nominal variables. For continues variables the Mann–Whitney U test was used. The diagnostic performance of sNGAL, uNGAL, the uNGAL/sNGAL ratio, the ratio between uNGAL and urine creatinine (uNGAL/uCreat) and the ratio between uKIM-1 and urine creatinine (uKIM-1/uCreat) was reported as sensitivity, specificity, positive, and negative predictive value for AKI with their corresponding 95 % confidence intervals (with the use of MedCalc version 15.6). For each parameter, a receiver operating characteristics (ROC) curve was constructed as a summary statistic and the area under the ROC curve (AUROC) and its corresponding 95 % confidence intervals were calculated. The AUROC of each of the variables was compared to that of reference comparator variable serum creatinine in a pair-wise comparison using the method of Hanley and McNeil [28]. Optimal cut-off points were determined using the Youden index. All reported P values are two-tailed. P values less than 0.05 were considered statistically significant.

## Results

### Patient characteristics and AKI at presentation and during admission

During the study period, 50 patients with *P. falciparum* malaria were included in the Rotterdam Malaria Cohort. Ten of these patients met the criteria for severe malaria and seven had AKI. Of 39 of these patients, including the ten patients with severe malaria and six of the patients with AKI, residual urine and serum samples were available for analysis. The general characteristics on admission of these 39 patients are shown in Table 1. Six patients (15 %) developed AKI, defined as KDIGO stage 1, 2 or 3, in the course of their infection. In four of these patients (67 %) AKI was already identified on admission; one patient was classified in KDIGO stage 1, two in stage 2 and one in stage 3. Two of these patients later progressed to a more advanced KDIGO stage. Two patients did not meet the KDIGO criteria at presentation but progressed during admission to KDIGO stage 3 (see Table 2).

**Table 1:** General characteristics

	AKI n=6	No AKI n=33	P value
*Demographic findings*			
Age, years	43 (29–62)	46 (28–69)	0.89
Male gender	3 (50 %)	30 (91 %)	<0.05
Ethnicity			1.00
Caucasian	2 (33 %)	9 (27 %)	1.00
African	4 (67 %)	22 (67 %)	1.00
Asian	0 (0 %)	2 (6 %)	1.00
Adequate prophylaxis use	0 (0 %)	0 (0 %)	1.00
Symptoms ≥8 days	2 (33 %)	6 (19 %)	0.58
Nephrotoxic co-medication^a^	1 (17 %)	4 (12 %)	1.00
*Clinical findings*			
Temperature °C	39.5 (38.3–40.1)	38.4 (35.5–40.7)	0.11
Systolic blood pressure, mmHg	100 (73–140)	125 (95–170)	0.08
Pulse rate beats/minute	111 (100–140)	95 (56–129)	<0.05
Glasgow coma scale	15 (15–15)	15 (11–15)	0.54
*Laboratory findings*			
Creatinine, µmol/L	196 (77–1081)	96 (39–133)	<0.01
Urea, mmol/L	14.8 (9.1–55.8)	5.6 (2.7–33.6)	<0.001
Sodium, mmol/L	129 (120–136)	137 (127–141)	<0.01
Potassium, mmol/L	3.2 (2.9–3.9)	3.7 (3.3–4.5)	<0.05
Lactate, mmol/L	4.5 (1.9–5.8)	1.5 (0.6–4.6)	<0.001
Parasitaemia, parasites/µL	386,600 (45,900–1,380,600)	39,200 (64–678,400)	<0.05
sNGAL, ng/ml	700 (174–2287)	91 (19–204)	<0.001
uNGAL, ng/ml	5792 (376–22,028)	9 (1–240)	<0.001
uKIM-1, ng/ml	3.9 (1.8–7.8)	1.2 (0.1–8.7)	<0.01
uNGAL/ sNGAL	10.1 (0.4–25.1)	0.1 (0.0–2.6)	<0.001
uNGAL/uCreatinine	815.0 (5.4–2172.7)	0.8 (0.1–42.5)	<0.001
uKIM-1/uCreatinine	0.04 (0.02–0.08)	0.1 (0.0–2.9)	0.04
Concomitant bacterial infection	1 (17 %)	0 (0 %)	0.15
*Outcome*			
Severe malaria (WHO 2014)	5 (83 %)	5 (15 %)	<0.01
Renal replacement therapy	3 (50 %)	0 (0 %)	<0.01
ICU admission	4 (67 %)	4 (12 %)	<0.05
Death	1 (17 %)	0 (0 %)	0.15

Continuous variables are given as median (range). Nominal variables are given as number (percentage)

P values <0.05 are considered significant

^a^Self-reported use of non-steroidal anti-inflammatory drugs (NSAIDs), ACE-inhibitors, diuretics or lithium

**Table 2:** KDIGO stage at initial presentation and during admission in relation to serum and urine NGAL and urine KIM-1 for all six patients with AKI

KDIGO stage at presentation	Highest KDIGO stage during admission	RRT^a^	Serum NGAL (≤177 ng/ml)	Urine NGAL (≤72 ng/ml)	Urine KIM-1 (≤5.33 ng/ml)
0	3	Yes	*899*	*22,028*	1.84
0	3	No	*501*	*8691*	3.88
1	2	No	174	*376*	4.40
2	2	No	*2287*	*6452*	3.92
2	3	Yes	*205*	*5132*	3.87
3	3	Yes	*2014*	*838*	*7.75*

Italics: Elevated laboratory measurements

Reference ranges in brackets

^a^Renal replacement therapy

The non-AKI group contained more men than de AKI group but other demographic findings did not differ significantly. Pulse rate, parasite load and serum concentrations of creatinine, urea, lactate, sNGAL, uNGAL and uKIM-1 were significantly higher in the AKI group. Serum sodium and potassium concentrations were significantly lower in the AKI group. The ratios uNGAL/sNGAL and of uNGAL/uCreat were significantly higher in the AKI patients. Intensive care unit admission was significantly more common in the AKI group. Since AKI is one of the defining criteria for severe malaria [23] severe malaria was also more common in the AKI group.

### Identification of AKI by NGAL and KIM-1

Five out of six patients with AKI presented with elevated sNGAL. uNGAL was elevated in all six (Table 2). In contrast, only one of the AKI patients showed elevated uKIM-1 at presentation. All patients that were classified as KDIGO stage 3, either at presentation or later during admission, already showed elevated concentrations of both sNGAL and uNGAL at presentation.

In seven patients, high concentrations of sNGAL, uNGAL or uKIM-1 on admission were found despite a seemingly normal kidney function throughout their admission; one had elevated sNGAL, uNGAL and uKIM-1, two patients showed elevated sNGAL and uNGAL with normal uKIM-1, three patients presented with only an elevated uNGAL, and in one patient only uKIM-1 was elevated. These data are available as Additional file 4.

### Diagnostic performance of sNGAL, uNGAL and uKIM-1 at presentation for AKI

Analysis of the diagnostic accuracy of sNGAL, uNGAL, uKIM-1 and the ratios of uNGAL and uKIM-1 to to urine creatine was performed by pair-wise comparison of the AUROCs of these parameters to the AUROC of serum creatinine. All parameters showed large AUROCs. However, none of these differences were statistically significant when compared to the AUROC of creatinine, likely due to the small sample size of this study. uNGAL was found to have an excellent performance with PPV of 1.00 (95 % CI 0.54–1.00), an NPV of 1.00 (95 % CI 0.89–1.00) and an AUROC of 1.00 (95 % CI 1.00–1.00; Table 3). The ROC curves of serum creatinine, sNGAL, uNGAL, uKIM-1 are available as a Additional file 5.

**Table 3:** Descriptive statistics of diagnostic accuracy of various biomarker measurements at presentation for the presence or subsequent development of acute kidney injury (AKI)

Parameter and cut-off value	Sensitivity	Specificity	PPV	NPV	Youden index	AUROC	P value*
sCreatinine ≥ 128 µmol/L	0.83 (0.36–1.00)	0.97 (0.84–1.00)	0.83 (0.36–0.99)	0.97 (0.84–1.00)	0.80	0.85 (0.60–1.00)	
sNGAL ≥ 168 ng/ml	1.00 (0.54–1.00)	0.88 (0.72–0.97)	0.60 (0.26–0.88)	1.00 (0.88–1.00)	0.88	0.98 (0.93–1.0)	0.389
uNGAL ≥ 308 ng/ml	1.00 (0.54–1.00)	1.00 (0.89–1.00)	1.00 (0.54–1.00)	1.00 (0.89–1.00)	1.00	1.00 (1.00–1.00)	0.299
uNGAL/sNGAL ≥ 1.9	0.83 (0.36–1.00)	0.97 (0.84–1.00)	0.83 (0.36–1.00)	0.96 (0.84–1.00)	0.80	0.95 (0.85–1.00)	0.544
uNGAL/uCreat ≥ 5.1 ng/mmol	1.00 (0.54–1.00)	0.88 (0.72–0.97)	0.60 (0.26–0.88)	1.00 (0.88–1.00)	0.88	0.98 (0.93–1.00)	0.390
uKIM-1 ≥ 1.83 ng/ml	1.00 (0.54–1.00)	0.72 (0.54–0.87)	0.40 (0.16–0.68)	1.00 (0.86–1.00)	0.72	0.87 (0.75–0.99)	0.919
uKIM-1 /uCreat ≤ 0.085 ng/mmol	1.00 (0.54–1.00)	0.58 (0.39–0.74)	0.30 (0.12–0.54)	1.00 (0.82–1.00)	0.58	0.76 (0.61–0.92)	0.810

Data are given as mean (95 % confidence interval)

Optimal cut-off values were determined using the Youden index

*PPV* positive predictive value; *NPV* negative predictive value; *AUROC* area under the ROC curve

* P values of pair-wise comparison of AUROCs are given (with creatinine ROC curve as comparator)

### Diagnostic performance of sNGAL, uNGAL and uKIM-1 at presentation for KDIGO stage 3

Analysis of the diagnostic accuracy of creatinine, NGAL and KIM-1 for the most severe form of AKI, KDIGO stage 3, was also performed. uNGAL (0.99, 95 % CI 0.88–1.0) and sNGAL (0.97, 95 % CI 0.86–1.00) were found to perform excellently and all evaluated parameters had larger AUROCs than creatinine (AUROC of 0.75, 95 % CI 0.59–0.87). Differences were not significant, but sample sizes in this sub-group were very small (see Additional file 6).

### Renal replacement therapy (RRT) in relation to sNGAL, uNGAL and uKIM-1 at presentation

Three patients developed a need for RRT. One of these patients did not classify as AKI on admission but later progressed to KDIGO stage 3, one was in KDIGO stage 2 and one in stage 3. However, all three presented with elevated sNGAL and uNGAL and one also showed an elevated urine KIM-1 on admission.

### Follow-up measurements during admission

For two patients, sNGAL and uNGAL concentrations were available for follow-up during admission. One patient needed RRT, the other did not. Both patients showed elevated uNGAL at initial presentation, while serum creatinine concentrations were still normal. No major changes of sNGAL concentrations were seen during admission (see Additional file 7).

## Discussion

In the present pilot study it is demonstrated that sNGAL and uNGAL have an excellent predictive performance for malaria-induced AKI.

Biomarkers for the early detection of AKI are under intense investigation and studies have revealed promising results in a broad variety of settings including contrast-induced nephropathy, critically ill patients, cardiac surgery, and kidney transplantation [29–35]. In several studies, both urine and serum NGAL concentrations were found to be superior to the serum creatinine concentration for the prediction of AKI [33, 36].

Early detection of AKI in malaria could help to prevent further deterioration of kidney function by appropriate fluid management, avoidance of nephrotoxic drugs and close monitoring to assess the need for RRT [3, 4, 37]. However, very few studies on biomarkers for AKI have been performed in malaria. The value of uNGAL was previously studied in a cohort of adult patients with severe *P. falciparum* infection in Bangladesh. uNGAL was shown to increase with increasing severity of AKI [2], but creatinine was found to be superior to uNGAL in predicting the need for RRT [15]. However, patients in this study generally presented severely ill and more than half of them already had a decreased eGFR on admission. One could speculate that the benefit of an early marker for AKI can be larger in the very different population of travellers with imported malaria, who tend to present in a much earlier stage of the infection.

In the present pilot study on biomarkers of AKI in imported malaria, highly promising results were observed for uNGAL, which was able to identify all six AKI patients at initial presentation. Follow-up measurements in two AKI patients revealed that uNGAL increases before serum creatinine starts to rise (Additional file 7). The AUROCs of sNGAL, uNGAL and uKIM-1 were large but, due to the very small sample size, the study was underpowered to demonstrate statistically significant differences. This is a major limitation of the present study and larger studies should be performed to demonstrate whether these biomarkers truly outperform serum creatinine.

The suggestion that uNGAL performs better than sNGAL, is an important discussion in literature. A meta-analysis conducted by Haase et al. showed that both serum and urine NGAL concentrations appear to perform similarly well and provide a relevant advantage as compared with serum creatinine [13]. It deserves further investigation whether this conclusion also applies to the performance of serum and urine NGAL in imported *P. falciparum* malaria. These results are important since oliguria is a common event in malaria-induced AKI [37] and the use of urine NGAL for diagnosis of malaria-induced AKI, therefore, has its limitations. Another ongoing debate is whether urine biomarker concentrations are better reported as absolute values or as a ratio to creatinine concentration [38]. In the present study, the absolute values seemed to perform somewhat better than the ratio.

Although uKIM-1 performed well when the cut-off level was set at 1.83 ng/ml, only one of the six patients with AKI had a uKIM-1 level above the upper limit of normal, which is 5.33 ng/ml, while all six patients had markedly raised uNGAL levels. The patient who did have an elevated uKIM-1 was already in KDIGO stage 3 at presentation. These findings raise the question whether the rise of KIM-1 levels occurs later in the course of early AKI than NGAL. A study in children undergoing cardiac surgery suggests that this might indeed be the case [39]. However, this study was performed in a study population, which was much different from the present, and more research is needed to confirm this finding in other clinical settings.

Several patients were found to have elevated sNGAL, uNGAL or uKIM-1 but did not progress to a KDIGO stage 1 or higher. The clinical significance of increased NGAL concentrations without detectable changes in creatinine, previously interpreted as a shortcoming of the biomarker, has been found to indicate sub-clinical AKI and to be of prognostic importance in critically ill adult patients [40]. NGAL can be influenced by extra-renal production by neutrophils caused by factors like concomitant systemic inflammation or malignancy [41]. However, it is unlikely that such bias influenced the findings in the present study to a large degree as co-infection was ruled out and malignancy was considered unlikely in all patients. Follow-up of patients with elevated NGAL concentrations deserves further investigation, as this would increase insight into the correlation of NGAL and the resolution of AKI or the development of chronic kidney disease.

An important limitation of biomarkers like NGAL and KIM-1 is that their availability is usually limited to resource-rich settings where malaria is only seen as an imported disease; in the regions that carry the largest burden of malaria, laboratory diagnostics are often limited to basic measurements. Even in resource-rich settings, studies on the cost-effectiveness of these biomarkers are still to be done [42].

## Conclusion

This pilot study reveals good diagnostic performance of sNGAL, uNGAL and uKIM-1. Especially uNGAL was found to have an excellent predictive performance. Larger studies are needed to demonstrate whether these biomarkers are superior to serum creatinine as predictors for AKI in *P. falciparum* malaria.

## Additional files

10.1186/s12936-016-1516-y KDIGO criteria.
10.1186/s12936-016-1516-y Estimated baseline serum creatinine concentration.
10.1186/s12936-016-1516-y WHO criteria for severe malaria (2014).
10.1186/s12936-016-1516-y Descriptive statistics of diagnostic accuracy of various laboratory measurements at presentation for the presence or development of AKI–KDIGO stage 3.
10.1186/s12936-016-1516-y ROC-curves for serum creatinine, sNGAL, uNGAL and uKIM-1 for the prediction of AKI (Figure).
10.1186/s12936-016-1516-y Dataset (Excel file).
10.1186/s12936-016-1516-y The course of the serum creatinine, serum NGAL and urine NGAL concentrations for two patients with AKI during admission (Figure).

## Abbreviations

AKI: acute kidney injury
KDIGO: Kidney Disease: Improving Global Outcomes
GFR: glomerular filtration rate
NGAL: neutrophil gelatinase-associated lipocalin
KIM-1: kidney injury molecule-1
sNGAL: serum neutrophil gelatinase-associated lipocalin
uNGAL: urine neutrophil gelatinase-associated lipocalin
uKIM-1: urine kidney injury molecule-1
QBC: quantitative buffy coat
RDT: rapid diagnostic test
eGFR: estimated glomerular filtration rate
MDRD: modification of diet in renal disease
WHO: World Health Organization
ROC: receiver operating characteristics
AUROC: area under the receiver operating characteristics curve
PPV: positive predictive value
NPV: negative predictive value
RRT: renal replacement therapy
