# Supplementary material for: Neutrophil gelatinase-associated lipocalin (NGAL) predicts the occurrence of malaria-induced acute kidney injury
Source: Malar J. 2016 Sep 9;15(1):464. doi: 10.1186/s12936-016-1516-y (PMC5017124; doi:10.1186/s12936-016-1516-y)
Supplement: Supplementary file 2 — 10.1186/s12936-016-1516-y Estimated baseline serum creatinine concentration. [file 12936_2016_1516_MOESM2_ESM.docx]

**Additional file 2: Estimated baseline serum creatinine concentration**

| **Age (years)** | **African ethnicity, males**  **μmol/L** | **Other males**  **μmol/L** | **African ethnicity, females**  **μmol/L** | **Other females**  **μmol/L** |
| --- | --- | --- | --- | --- |
| 20–24 | 133 | 115 | 106 | 88 |
| 25–29 | 133 | 106 | 97 | 88 |
| 30–39 | 124 | 106 | 97 | 80 |
| 40–54 | 115 | 97 | 88 | 80 |
| 55–65 | 115 | 97 | 88 | 71 |
| >65 | 106 | 88 | 80 | 71 |
| Estimated glomerular filtration rate = 75 (mL/min per 1.73 m^2^) = 186 × (serum creatinine [*s*Cr]) - 1.154 × (age) - 0.203 × (0.742 if female) × (1.210 if black) = exp(5.228 - 1.154 × In [*s*Cr]) - 0.203 × In(age) - (0.299 if female) + (0.192 if black). Adapted from Bellemo *et al*. [26] | | | | |
