# Supplementary material for: Neutrophil gelatinase-associated lipocalin (NGAL) predicts the occurrence of malaria-induced acute kidney injury
Source: Malar J. 2016 Sep 9;15(1):464. doi: 10.1186/s12936-016-1516-y (PMC5017124; doi:10.1186/s12936-016-1516-y)
Supplement: Supplementary file 4 — 10.1186/s12936-016-1516-y Descriptive statistics of diagnostic accuracy of various laboratory measurements at presentation for the presence or development of AKI–KDIGO stage 3. [file 12936_2016_1516_MOESM4_ESM.docx]

**Additional file 4: Descriptive statistics of diagnostic accuracy of various laboratory measurements at presentation for the presence or development of AKI - KDIGO stage 3**

| **Parameter and cut-off value^** | **Sensitivity** | **Specificity** | **PPV** | **NPV** | **Youden index** | **AUROC** | **P-value*** |
| --- | --- | --- | --- | --- | --- | --- | --- |
| sCreatinine  ≥ 124 µmol/L | 0.75  (0.19-0.99) | 0.91  (0.77- 0.98) | 0.50  (0.12-0.88) | 0.97  (0.84-1.00) | 0.66 | 0.75  (0.59-0.87) |  |
| sNGAL  ≥ 204 ng/ml | 1.00  (0.40-1.00) | 0.97  (0.85-1.00) | 0.80  (0.28-1.00) | 1.00  (0.90-1.00) | 0.97 | 0.97  (0.86-1.00) | 0.199 |
| uNGAL  ≥ 376 ng/ml | 1.00  (0.40-1.00) | 0.97  (0.85-1.00) | 0.80  (0.28-1.00) | 1.00  (0.90-1.00) | 0.97 | 0.99  (0.88-1.0) | 0.170 |
| uNGAL/sNGAL  ≥ 2.8 | 0.75  (0.19-0.99) | 1.00  (0.90-1.00) | 1.00  (0.29-1.00) | 0.97  (0.86-1.00) | 0.75 | 0.92  (0.78-0.98) | 0.359 |
| uNGAL/uCreat  ≥ 42.5 ng/mmol | 1.00  (0.40-1.00) | 0.97  (0.85-1.00) | 0.80  (0.28-1.00) | 1.00  (0.90-1.00) | 0.97 | 0.98  (0.87-1.0) | 0.173 |
| uKIM-1  ≥ 1.82 ng/ml | 1.00  (0.40-1.00) | 0.68  (0.50-0.83) | 0.27  (0.08-0.55) | 1.00  (0.85-1.00) | 0.74 | 0.82  (0.67-0.93) | 0.679 |
| uKIM-1 / uCreat  ≤ 0.54 ng/mmol | 1.00  (0.40-1.00) | 0.66  (48-81) | 0.25  (0.07-0.52) | 1.00  (0.85-1.00) | 0.66 | 0.85  (0.70-0.94) | 0.603 |
| Data are given as mean (95% confidence interval). Optimal cut-off values were determined using the Youden index. PPV = positive predictive value, NPV = negative predictive value, AUROC = Area Under the ROC curve. ***P-values of pair-wise comparison of Area Under ROC curves are given (with creatinine ROC curve as comparator). | | | | | | | |
