# Supplementary figures and images for: Neutrophil gelatinase-associated lipocalin (NGAL) predicts the occurrence of malaria-induced acute kidney injury
Source: Malar J. 2016 Sep 9;15(1):464. doi: 10.1186/s12936-016-1516-y (PMC5017124; doi:10.1186/s12936-016-1516-y)

**Additional file 5: ROC- curves for serum creatinine, sNGAL, uNGAL and uKIM-1 for the prediction of AKI.**

**
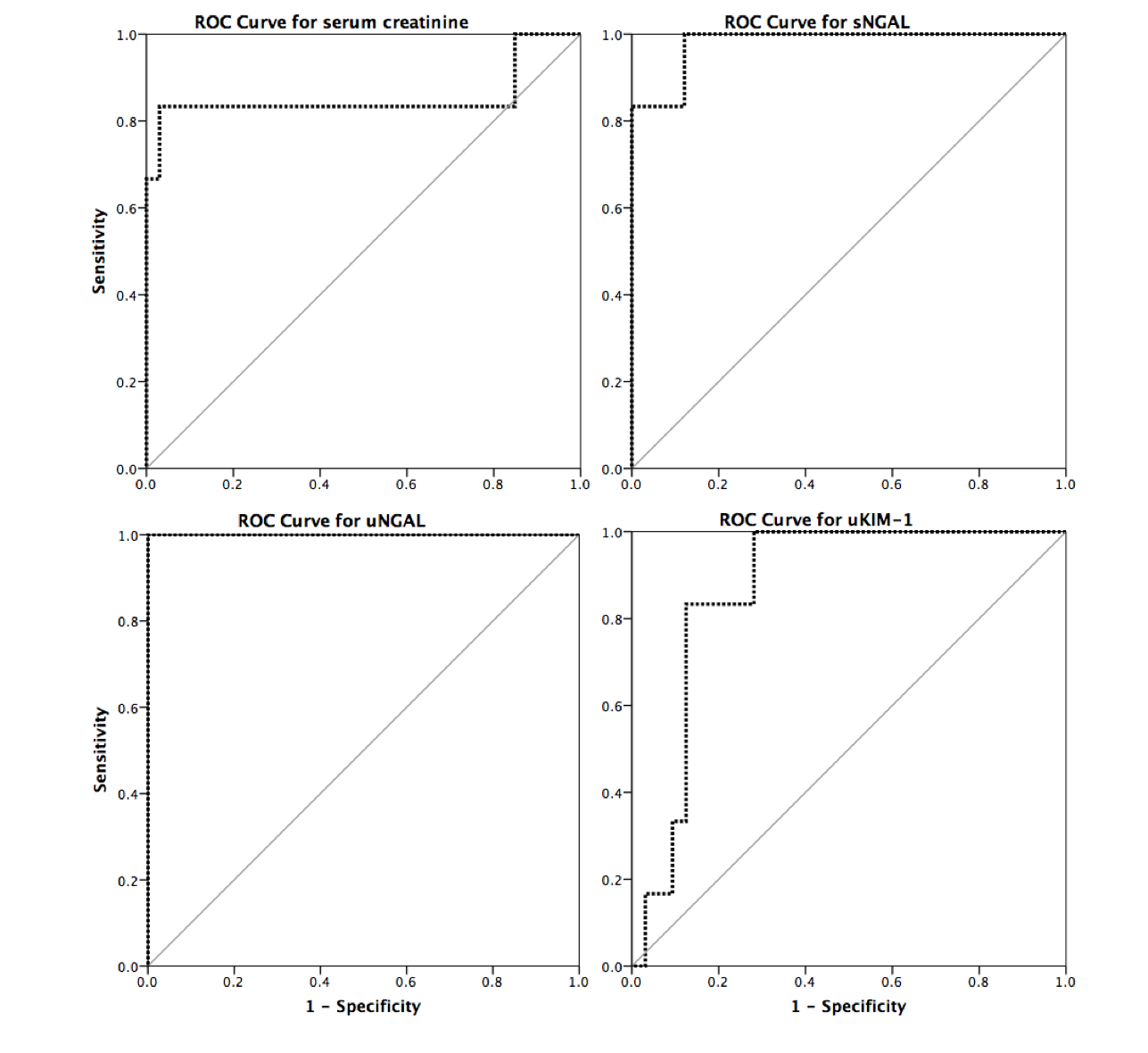
**

Supplement: Supplementary file 5 — 10.1186/s12936-016-1516-y ROC-curves for serum creatinine, sNGAL, uNGAL and uKIM-1 for the prediction of AKI (Figure). [file 12936_2016_1516_MOESM5_ESM.docx]
