# Supplementary material for: Neutrophil gelatinase-associated lipocalin (NGAL) predicts the occurrence of malaria-induced acute kidney injury
Source: Malar J. 2016 Sep 9;15(1):464. doi: 10.1186/s12936-016-1516-y (PMC5017124; doi:10.1186/s12936-016-1516-y)
Supplement: Supplementary file 7 — 10.1186/s12936-016-1516-y The course of the serum creatinine, serum NGAL and urine NGAL concentrations for two patients with AKI during admission (Figure). [file 12936_2016_1516_MOESM7_ESM.docx]

**Additional file 7: The course of the serum creatinine, serum NGAL and urine NGAL concentrations for two patients with AKI during admission.**

**
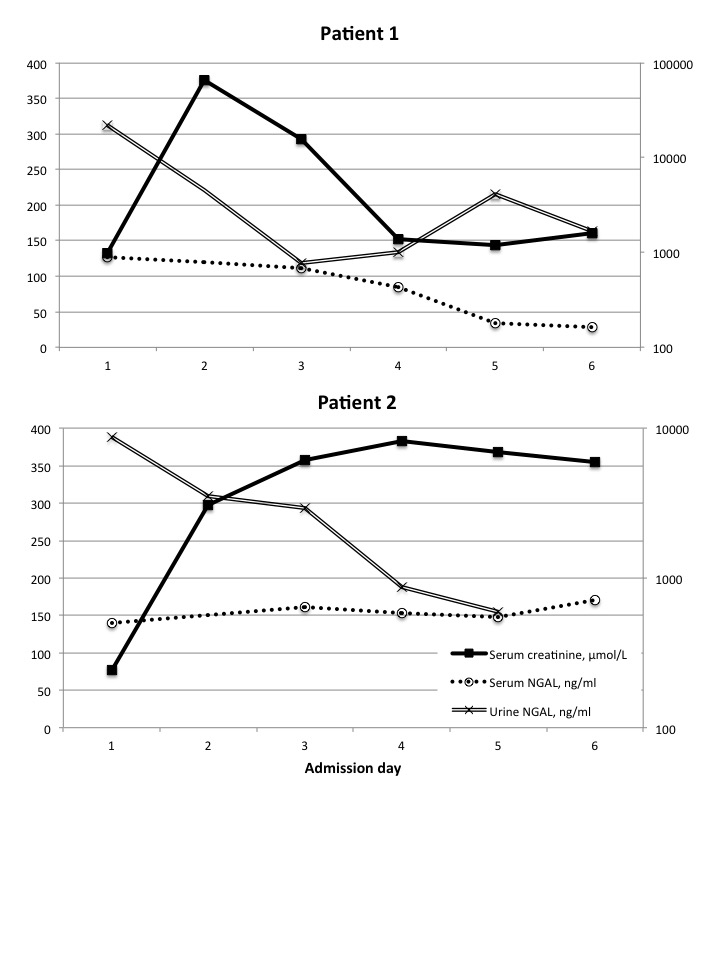
**
